# Some Remarks on the Effects of Drugs

**Published:** 1896-12

**Authors:** 


SOME REMARKS ON THE EFFECTS OF DRUGS. (From the yearly reports of the Bavarian veterinarians.) Bromocalium: Hohenleitner observed twelve cases of epilepsy in cattle, swine, and goats in which this drug was given with favorable results.

Cocaine: A valuable and spirited riding-horse had a tear 7 cm. long in the right nostril, but would not allow it to be sewed up. After bathing with a 15 per cent. solution of cocaine the horse allowed it to be sewed without resistance.

Iodocalium: Schwäbel cured three cases of actinomycosis of the tongue in cattle by the application of 8 grains iodocalium for ten to twelve days. King also obtained a complete healing in nine and a great improvement in three cases of actinomycosis by giving 6 to 12 grains of iodocalium per day. Engel also reports good results from the use of the same drug. Hohenleitner restricted himself to iodocalium in the treatment of actinomycosis. In spite of the fact that many cases did not yield to the treatment, he was, on the whole, satisfied with the results.—*Schweizer Archiv*, January and February, 1896.

